# Internal Brace Augmentation Following Ulnar Collateral Ligament Release for Elbow Stiffness: A Case Series

**DOI:** 10.3390/jcm15186973

**Published:** 2026-09-09

**Authors:** Marco Foreman, Supreeya Saengchote, Anton Khlopas, Morad Chughtai, Robert C. Matthias, Joseph J. King

**Affiliations:** 1 Department of Orthopaedic Surgery and Sports Medicine, University of South Florida Morsani College of Medicine, Tampa, FL 33612, USA; 2 College of Medicine, University of Florida, Gainesville, FL 32610, USA; 3 Department of Orthopedic Surgery, Henry Ford Health System, Detroit, MI 48202, USA; 4 Florida Orthopaedic Associates, DeLand, FL 32720, USA; 5 Department of Orthopaedics and Sports Medicine, University of Florida, Gainesville, FL 32607, USA

**Keywords:** elbow surgery, elbow contracture, elbow instability, internal brace augmentation, ulnar collateral ligament, ligamentous reconstruction

## Abstract

**Background:** Elbow stiffness is a common post-traumatic complication that may require surgical intervention in severe cases. Capsular and ulnar collateral ligament (UCL) release can restore motion but may result in iatrogenic valgus instability. Internal brace (IB) augmentation has emerged as a potential method to restore stability while preserving mobility. This study compares the utilization of IB augmentation following UCL release to conventional techniques. **Methods**: We report two cases of patients with severe post-traumatic elbow contractures and associated heterotopic ossification (HO). Both underwent HO excision, capsular release, and UCL release or excision followed by IB augmentation. Outcomes were assessed using range of motion (ROM), visual analog scale (VAS) pain scores, and radiographic evaluation at follow-up. **Results:** In Case 1, bilateral elbows improved from near ankylosis preoperatively to final arcs of motion from −15° extension to 120° flexion (left) and −15° to 125° (right), with VAS improving to 0. In Case 2, motion improved from −45° extension to 90° flexion preoperatively to −45° to 108° at final follow-up, with a VAS of 0. No valgus instability, subluxation, or HO recurrence was observed in either case. **Conclusions:** IB augmentation following UCL release is feasible and may provide effective stabilization while enabling meaningful improvements in elbow motion. Larger studies are needed to further evaluate its clinical utility and durability.

## 1. Introduction

Anatomically, the elbow is a synovial hinge joint whose motion is essential for upper extremity function to position the hand in space [1]. Due to the elbow joint’s distinctive anatomy, i.e., the presence of three separate articulations within a single synovial cavity, vulnerable soft tissue, and the proximity of the brachialis muscle to the capsule, it is prone to stiffness following both atraumatic and traumatic insults [2]. Elbow stiffness, also known as contracture, commonly occurs secondary to traumatic injuries such as high-energy fractures, thermal burns, and postoperative sequelae [3].

Elbow stiffness is a common post-traumatic complication with an incidence of at least 5% [4]. The pathogenesis of elbow stiffness affecting flexion is predominantly due to soft tissue contracture and/or osseus congruency disruption over the ulnar collateral ligament (UCL) of the affected joint [5]. Specifically, the most common extrinsic cause of elbow contracture is heterotopic ossification (HO) [6]. HO itself is predominantly the result of acute inflammation and local hypoxia, which together activate BMP, TGF-β, and HIF-1α signaling pathways that redirect tissue-resident mesenchymal progenitors away from normal repair and toward ectopic endochondral bone formation [7,8].

In practice, this can be treated through an arthroscopic anterior or posterior débridement, as well as open procedures [9]. However, in cases of severe contracture, the excision of HO bone, capsular release of the posterior band of the UCL, and even full release in some instances are required [10]. Occasionally, complete excision of the UCL is needed due to severe heterotopic ossification, which leaves no soft tissue remaining for repair. Although UCL release can help effectively remedy the contracture, this technique has been described across the literature to result in an iatrogenically unstable elbow joint upon valgus load [11,12]. This complication is secondary to the medial collateral ligament’s essential role of providing static and dynamic resistance to valgus and rotational stresses [11,12].

Several techniques can be used in clinical practice to address this pathologic valgus laxity, including UCL reconstruction, dynamic or static external fixation, and placement of an internal joint stabilization device. Although these conventional procedures have historically reported positive clinical outcomes, they pose the risk of several complications, with some requiring staged procedures and reoperation. One systematic review quoted a 73% complication rate for open arthrolysis and external fixator placement for post-traumatic elbow stiffness [13]. The purpose of this study is to present two cases of severe elbow contracture with UCL heterotopic ossification managed by capsular release and novel internal brace (IB) augmentation by a single surgeon at one institution. The patients were selected based on their analogous traumatic mechanism of injury, subsequent contracture severity, and presence of severe heterotopic ossification of the UCL. We also review the advantages, disadvantages, and outcomes of IB augmentation versus conventional management.

## 2. Cases

Case 1: A 56-year-old right-hand-dominant male with a history of alcohol abuse and depression presented with limited elbow ROM due to bilateral upper extremity contractures from third-degree burns in 2019. He subsequently underwent multiple débridements and skin grafts on both arms. Upon evaluation, physical exam demonstrated full pronation and supination of the right forearm, pronation to 80° and neutral supination on the left, and complete loss of flexion and extension arcs in both elbows. Sensation was intact to light touch in the median, ulnar, and radial nerve distributions, though he reported altered sensation in the volar small fingers bilaterally.

**Figure 1:**
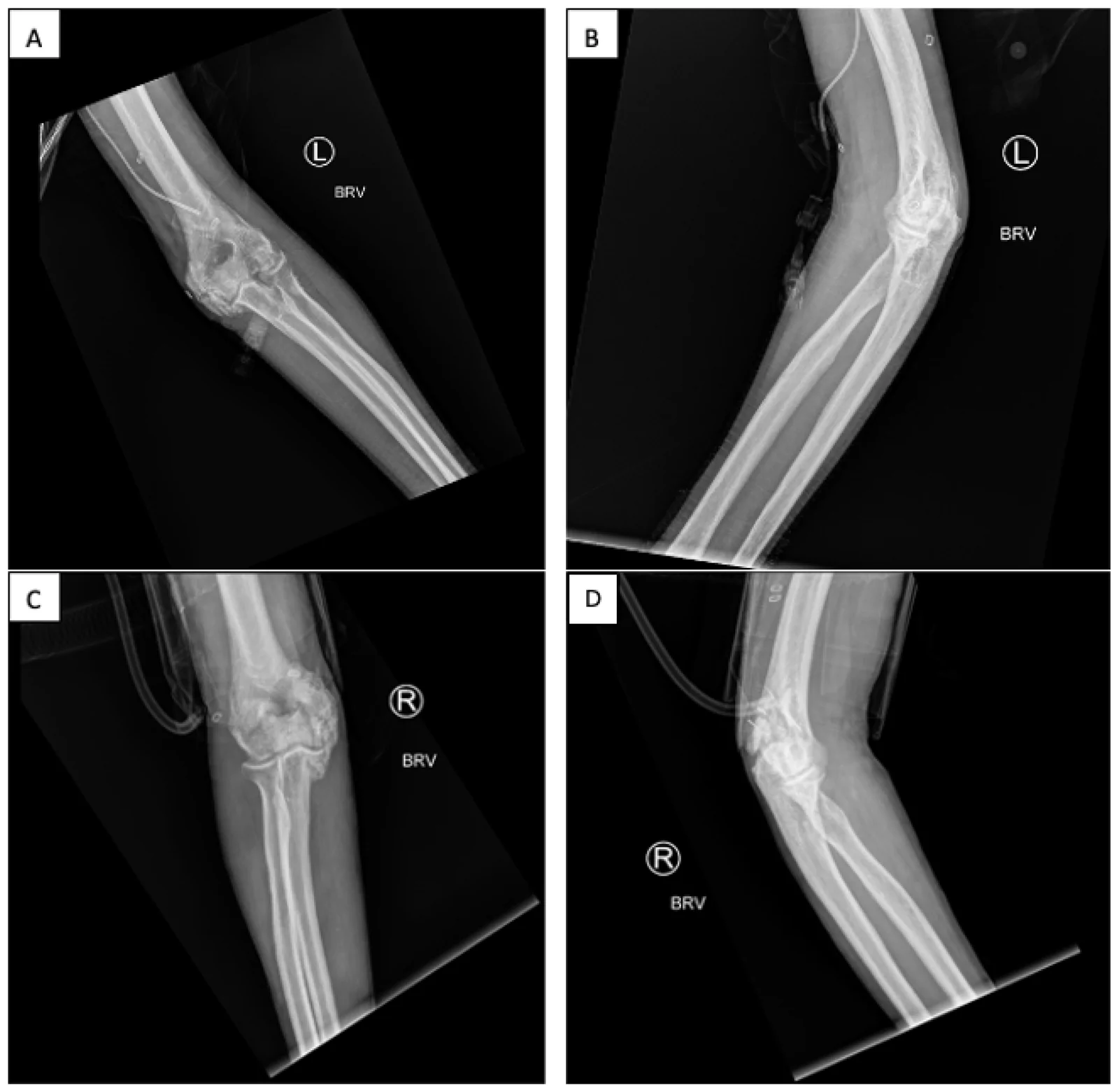
Preoperative (**A**,**C**) anterior to posterior (AP) radiographs of the left and right upper extremities, respectively. (**B**,**D**) Lateral radiographs of the left and right upper extremities, respectively. This figure demonstrates severe, bridging heterotopic ossification about the ulnohumeral joint bilaterally upon initial presentation.

**Figure 2:**
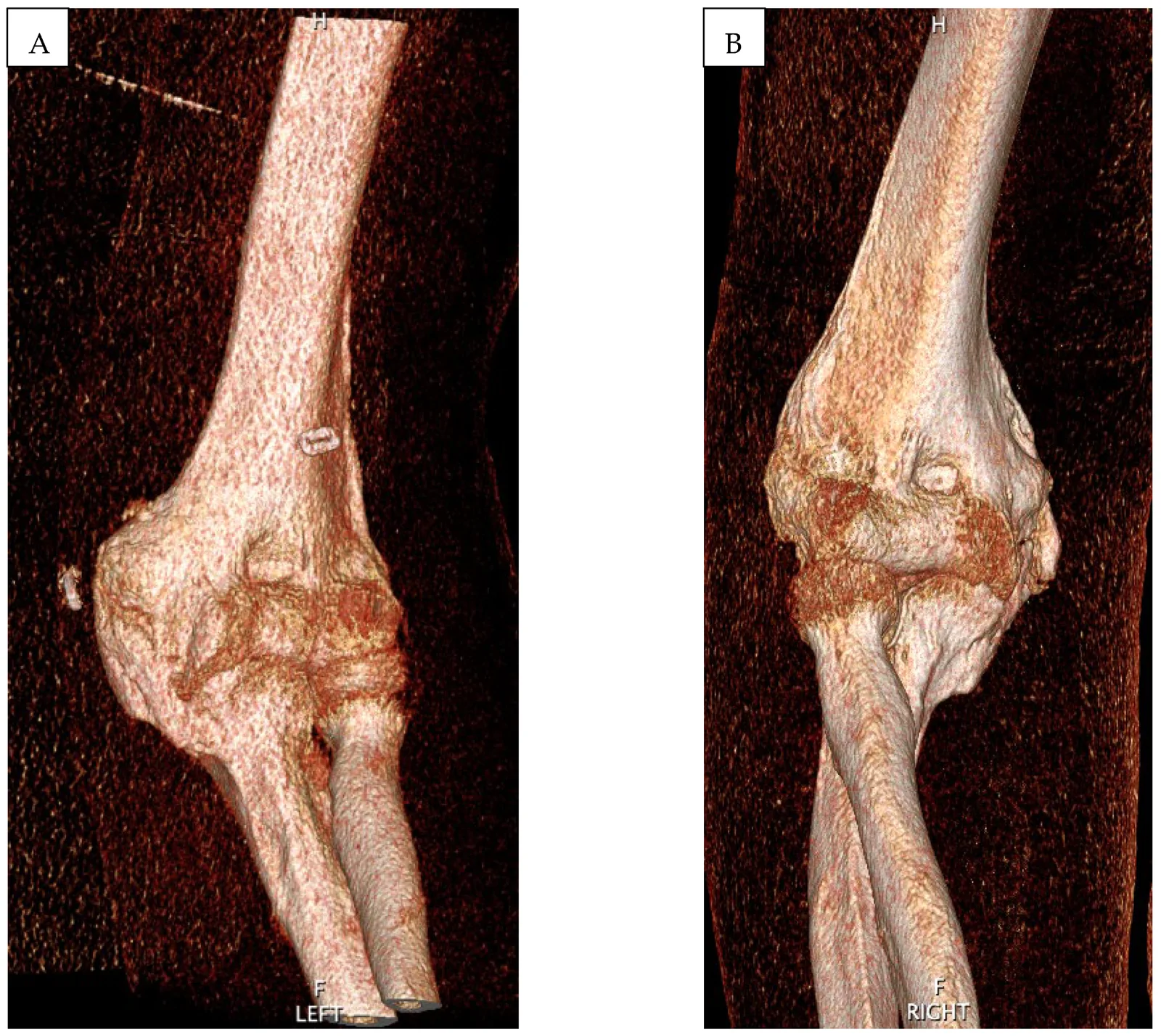
Preoperative (**A**,**B**) 3D computed tomography renderings demonstrating severe heterotopic ossification about the left and right ulnohumeral joints, respectively. These images were used preoperatively to guide treatment.

Radiographs of bilateral elbows revealed bridging HO about the ulnohumeral joint bilaterally (Figure 1). A computed tomography (CT) was obtained for preoperative planning, which demonstrated bridging of HO, notably posteromedial to the left elbow as well as medial and lateral to right elbow (Figure 2). The patient elected to proceed with surgical management. The operative plan was to address the patient’s left elbow first, beginning with prophylactic radiotherapy to prevent HO recurrence. At the time of surgery, the patient underwent left elbow capsular release and excision of heterotopic bone including resection of the ossified ulnar collateral ligament. Subsequently, medial IB fixation was performed due to subjective valgus instability with absence of a UCL following capsular release and heterotopic bone resection. The internal brace technique used here was a knotless anchor in the sublime tubercle with a Fibertape suture (Arthrex), and then a second knotless anchor with the Fibertape was placed in the isometric point on the distal aspect of the medial epicondyle. The sliding sutures were used to affix the remaining surrounding tissue to the medial epicondyle. Left ulnar nerve decompression at the elbow with subcutaneous ulnar nerve transposition was also performed. Subsequently, when ranging the patient’s elbow in the operating room, he achieved full extension and flexion to 120° with near full pronation and supination.

**Figure 3:**
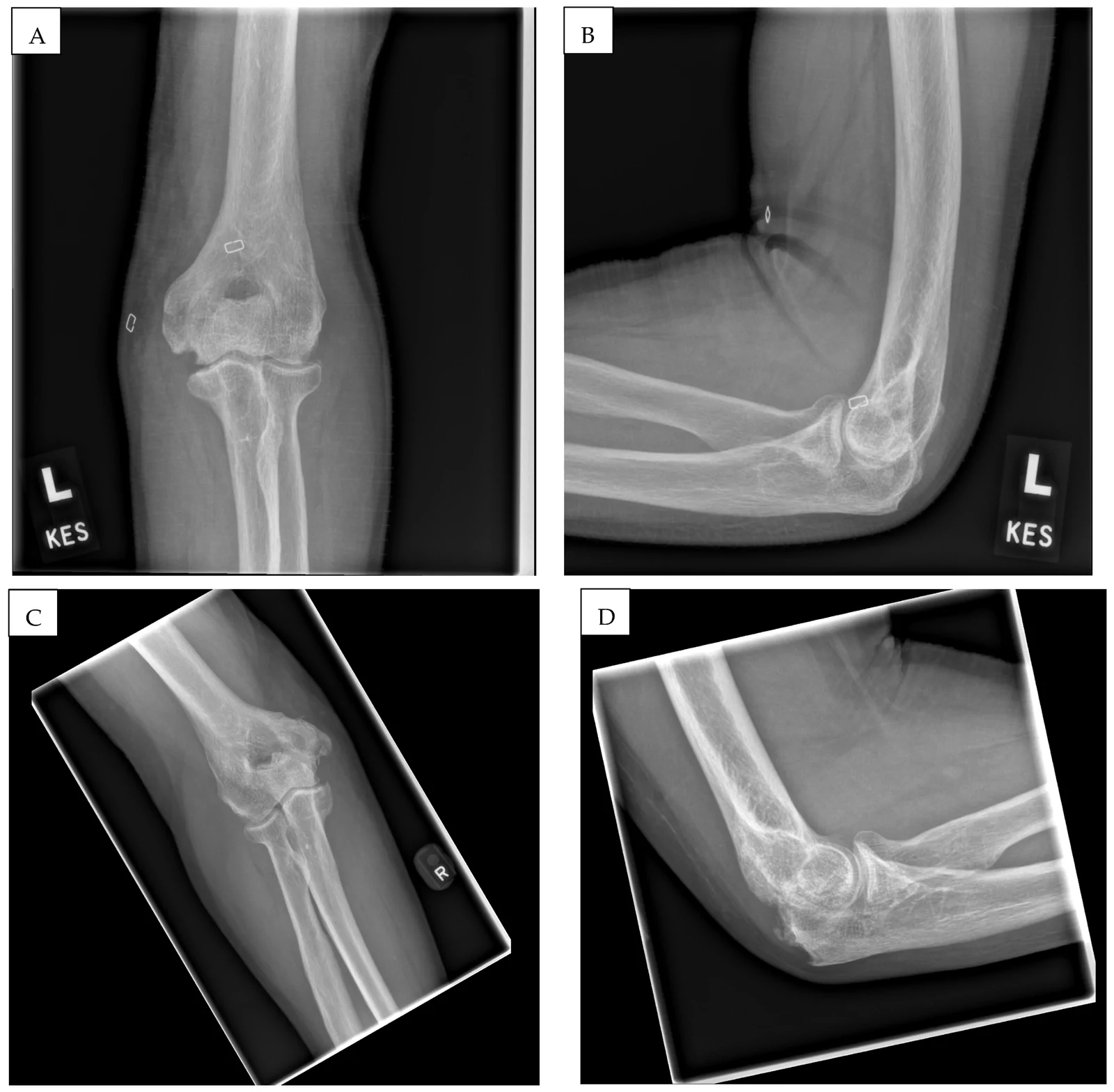
Postoperative (**A**,**C**) anterior to posterior (AP) radiographs of the left and right upper extremities, respectively. (**B**,**D**) Lateral radiographs of the left and right upper extremities, respectively. This figure demonstrates appropriate alignment, healing, and omission of abnormal bony overgrowth at final follow-up, bilaterally.

At one-week follow-up, the patient noted subjective improvement in his left elbow mobility and mild pain with a visual analog score (VAS) of 5. On physical exam, the patient had left elbow active flexion to 70°, extension to −15°, supination to 90°, and pronation to 45°. At two-month follow-up, left elbow active flexion was to 80° and extension to 10°, and an X-ray of the joint demonstrated no evidence of subluxation, dislocation, or HO recurrence. Final follow-up at two years demonstrated satisfactory improvement with active range of motion (AROM) −15° to 120° in extension and flexion, supination and pronation to 75° and 80°, respectively, and a VAS of 0. Notably, he had a stable elbow to varus and valgus stress, and radiography demonstrated no recurrence of HO (Figure 3).

The patient similarly underwent surgical management of his right elbow contracture with replication of the aforementioned steps. Intraoperatively, a final ROM of 10° to 110° in extension and flexion was achieved with full pronation and supination. At one-month follow-up, the patient had a VAS of 6 and right elbow extension and flexion arc of −30° to 65°. Three-month follow-up was notable for improvement in elbow extension and flexion arc of 20° to 90°, with VAS of 0. Final follow-up at one year demonstrated satisfactory results with AROM −15° to 125° extension and flexion, supination and pronation to 75° and 80°, and VAS of 0. Similarly, he had a stable elbow to varus and valgus stress, and radiography demonstrated no recurrence of HO (Figure 3).

**Figure 4:**
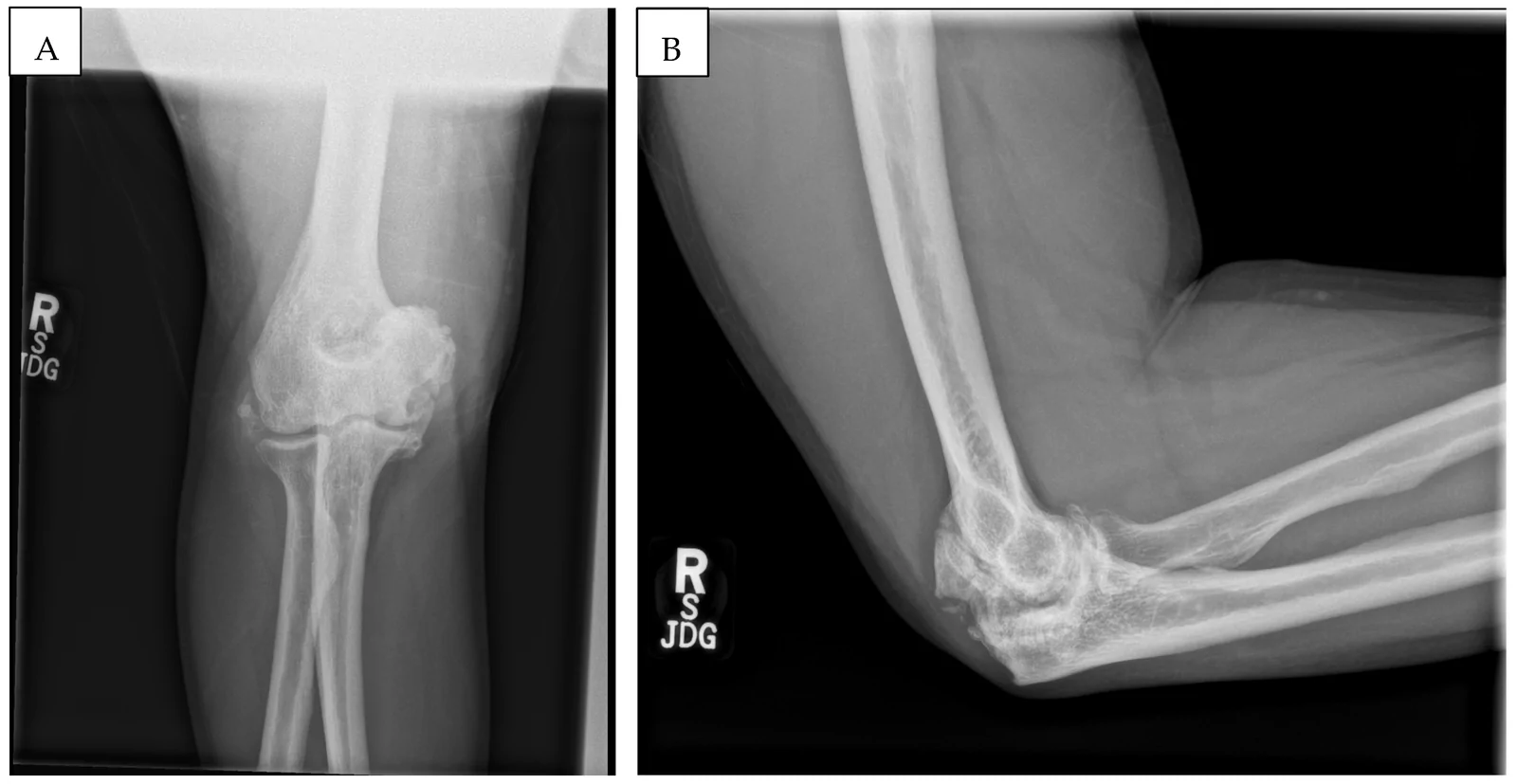
Preoperative (**A**) anterior to posterior (AP) radiograph. (**B**) Lateral radiograph. This figure demonstrates severe heterotopic ossification of the right upper extremity at the posteromedial aspect of the humeroulnar articulation upon initial presentation.

Case 2: A 41-year-old right-hand-dominant male with a history of type II diabetes and hypertension presented with persistent right elbow stiffness after a motorcycle accident in 2021. He had limited improvement in ROM despite months of physical and occupational therapy but remained pain-free without subjective paresthesia. Physical examination of the right elbow was notable for decreased ROM at −45° of extension to 90° of flexion, with supination and pronation to 80° and 85°, respectively. An X-ray of his right elbow demonstrated maturing HO about the posteromedial aspect of the humeroulnar articulation, consistent with chronic deformity of the olecranon process of the ulna (Figure 4).

**Figure 5:**
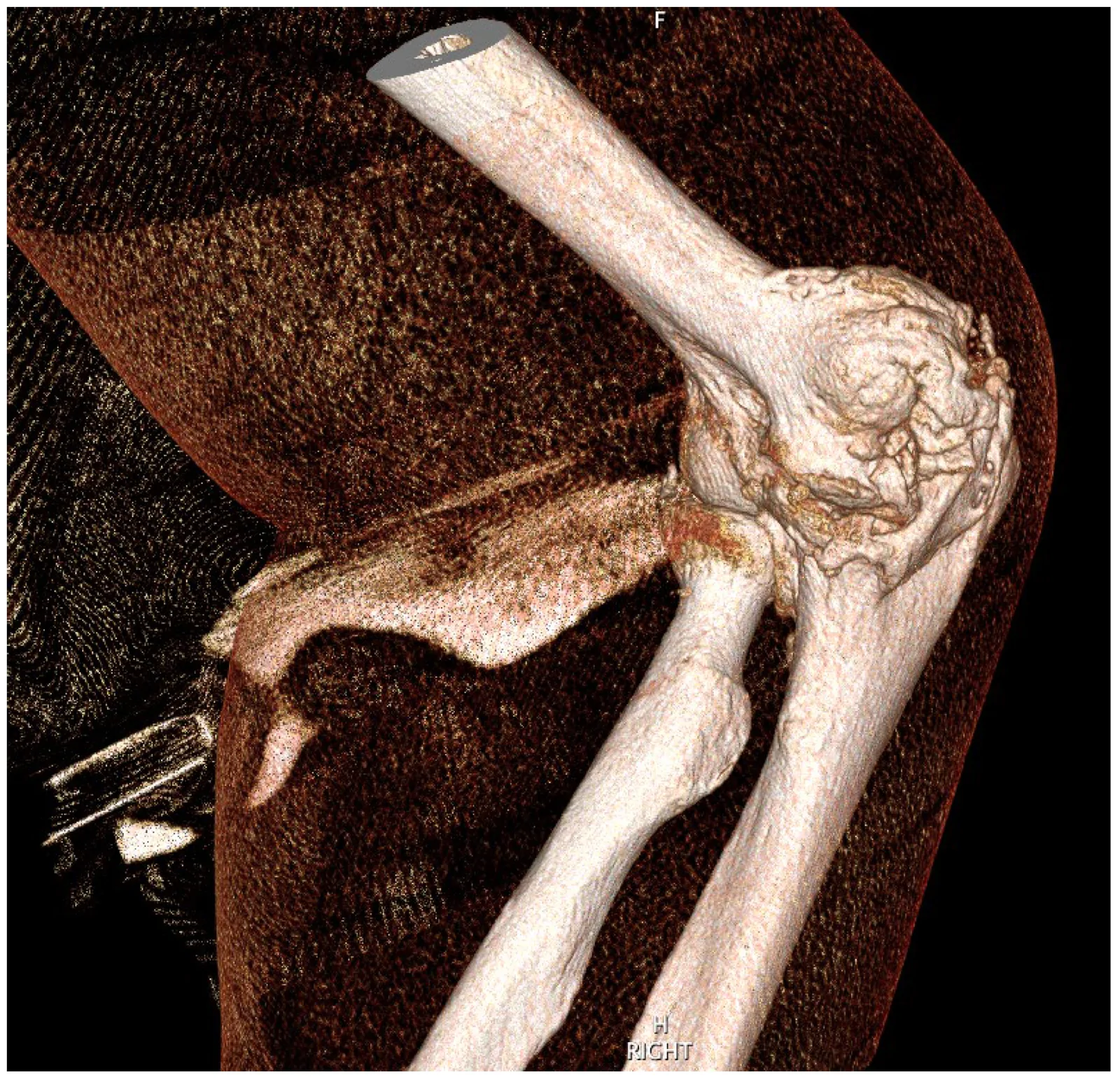
Preoperative 3D computed tomography rendering demonstrating severe heterotopic ossification with near complete bridging across the ulnar collateral ligament, lateral ulnar collateral ligament, and posteromedial aspect of the elbow. This image was used preoperatively to guide treatment.

A non-contrast CT of the right elbow was ordered for preoperative planning. The results of the CT demonstrated severe HO about the posterior bundle of the UCL and lateral ulnar collateral ligament (LUCL), with near complete bridging across the posteromedial aspect of the elbow (Figure 5). The patient elected to proceed with surgical management.

Prior to surgery, the patient underwent radiotherapy to prevent further HO formation. At the time of surgery, the patient underwent right elbow joint arthrotomy with capsular release, excision of heterotopic bone, right ulnar nerve decompression at the elbow with subcutaneous ulnar nerve transposition, and UCL augmentation with IB fixation given that the native ligament was ossified and therefore excised in this procedure due to subjective valgus instability at the time of surgery. When ranging the patient’s elbow in the operating room, an improved ROM from 15° of extension to 135° degrees of flexion was achieved, with full pronation and supination arcs.

**Figure 6:**
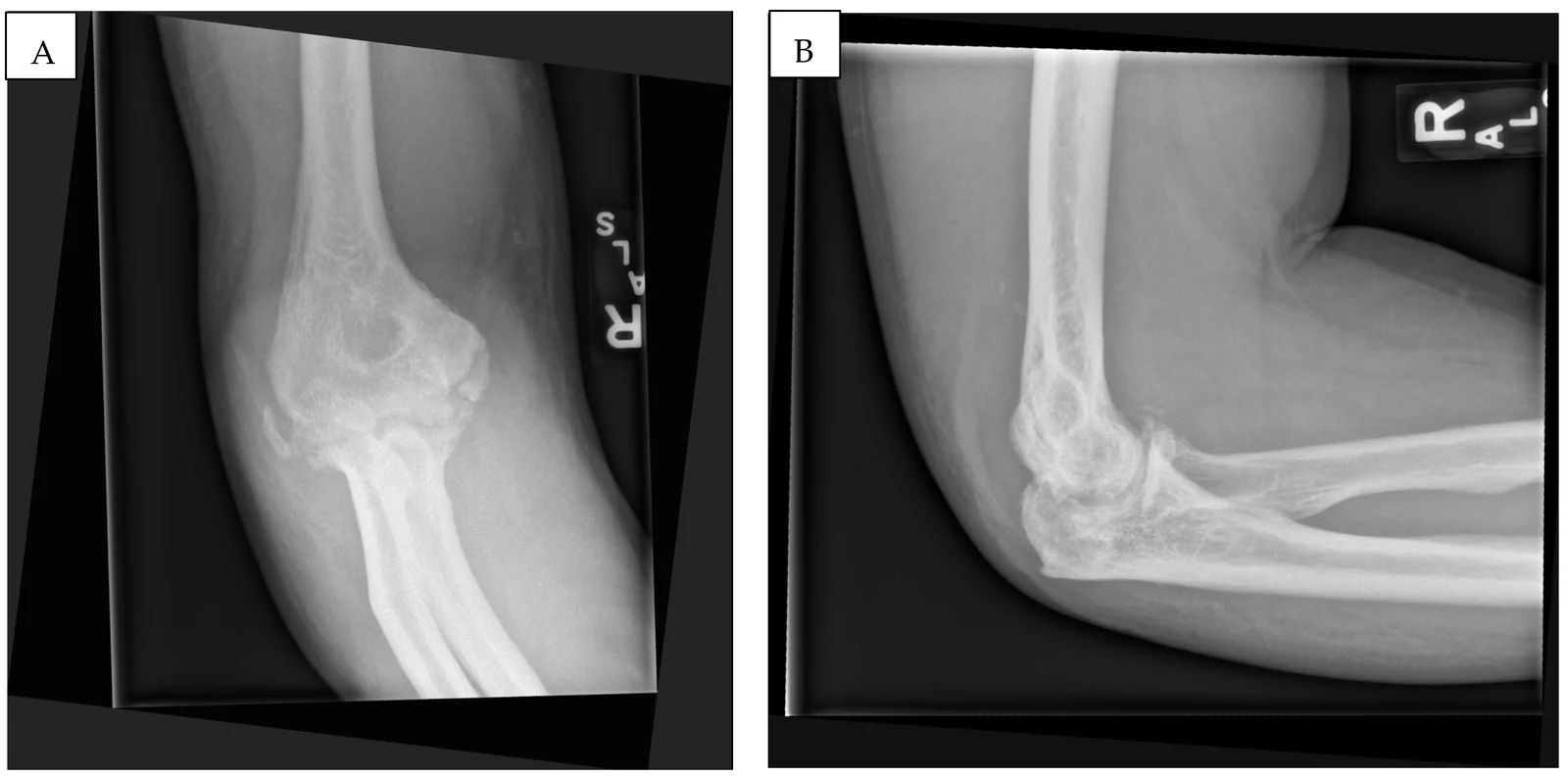
Postoperative (**A**) anterior to posterior (AP) radiograph. (**B**) Lateral radiograph. This figure demonstrates satisfactory elbow alignment and no new HO formation at final follow-up.

At one-week follow-up, the patient reported no pain, with a VAS of 0 and physical examination demonstrating AROM −54° to 96° of extension and flexion, with supination and pronation to 60° and 70°, respectively. Final follow-up at six weeks revealed an improved AROM −45° to 108° of extension and flexion, with pronation and supination to 30° and 30°, respectively. Additionally, he had a stable elbow to varus and valgus stress testing. Radiographs demonstrated satisfactory alignment with no further HO formation (Figure 6).

## 3. Discussion

Post-traumatic stiffness of the elbow is a frequent and challenging complication for both patients and surgeons due to the disabling nature of elbow contracture as well as the biomechanical hurdles posed when treating this pathology. Functionally, loss of terminal flexion is more disabling than loss of terminal extension and thus has been the target of both non-surgical and surgical interventions [14]. For this reason, especially for instances in which elbow contracture is recalcitrant to nonoperative methods, the goal of surgical management is to restore native or near-native flexion, commonly via arthroscopic anterior/posterior débridement, open arthrolysis, and—in severe cases—varying degrees of open UCL release [2]. Unfortunately, this latter technique often resolves flexion contracture at the expense of the elbow’s resistance to valgus load, ultimately predisposing the joint to iatrogenic instability and laxity.

### 3.1. Internal Brace Augmentation

This case series highlights two middle-aged males: one with second- and third-degree burns and another from a high-impact vehicular accident. Both developed severe elbow contractures from bridging HO at the proximal ulnohumeral joint, a common sequela of elbow trauma [15]. Following UCL release and IB augmentation, Case 1’s extension–flexion arc improved from complete bilateral contracture to −15° to 120° in the left arm and −15° to 125° in the right. Case 2’s range of motion improved from −45° extension to 90° flexion preoperatively to −45° extension to 108° flexion at the final follow-up. Notably, no instances of instability occurred, and a VAS of 0 was achieved in both cases. These cases of capsular release and UCL HO excision highlight the ability of an isolated UCL internal brace fixation without ligament repair or reconstruction to provide sufficient elbow stability.

Similarly, IB augmentation is increasingly used in UCL repair or reconstruction, particularly in throwing athletes prone to traumatic elbow injuries. UCL surgeries in New York state alone increased by 193% from 2002 to 2011 [16]. Several biomechanical studies have examined the potential benefits of IB via cadaveric elbow manipulation to assess the biokinetics of the ulnohumeral joint following various UCL repair strategies [16]. Notably, in the biomechanical studies comparing augmented IB versus conventional reconstruction techniques, it was found that IB restored valgus stability to the native state, with statistically significant improvements in torsional resistance, load capacitance, gap formation, and time-zero failure strength [17,18,19,20].

Of mention, there is a paucity of studies evaluating IB use in cases of iatrogenic instability following HO excision. A recent prospective case series evaluating IB for simple and complex elbow instability involved 34 patients treated with ligament bracing to augment repair, which yielded a mean ROM of 112° ± 29, an objective functional Mayo Elbow Performance Score (MEPS) of 88 ± 13 points, and 85% patient satisfaction [21]. According to Ellwein et al., these good clinical outcomes support the high primary stability findings purported by biomechanical studies and also allow for early functional mobilization—key aspects for facilitating postoperative rehabilitation [21]. Another study by Ozdag et al. analyzed 18 cases of acute and chronic elbow instability treated with UCL repair and IB augmentation between 2018 and 2020, with a mean follow-up of over 20 months [22]. The results of this study reported a mean flexion–extension arc of 124° and subjective patient metrics including Disabilities of the Arm, Shoulder, and Hand (DASH) and visual analog (VAS) pain scale scores of 17 and 2, respectively [22].

### 3.2. Conventional and Alternative Treatment Options

Conversely, conventional strategies to address UCL laxity upon arthrolysis and capsular release include collateral ligament reconstruction and ligamentous repair paired with external brace fixation. Regarding UCL reconstruction, the Modified Jobe (figure-of-eight) and docking techniques are the most common approaches. While the docking technique was traditionally favored for superior outcomes, recent studies show no significant difference. Both achieve excellent results, with 80–95% success rates in return to play, revision-free survival, and outcomes like the Kerlan–Jobe Orthopedic Clinic (KJOC) score and Conway Scale [23,24,25,26].

A more novel approach, first described by Dr. Camp and colleagues in 2019, the anatomic linear construct aims to recreate the native ulnar ligament insertion with greater tendon-to-bone contact than the Modified Jobe or docking techniques [27]. While biomechanically promising due to increased tendon-to-bone contact on both the ulnar and humeral sides and multipoint fixation for enhanced valgus stability, clinical outcomes are limited [28]. In addition to ligamentous repair, some surgeons elect to further stabilize the elbow with hinged external fixators. Two retrospective studies by Zhou et al. and Wang et al. evaluated outcomes of radical UCL release combined with ligament repair and hinged external fixators for post-traumatic elbow stiffness [29,30]. Both studies demonstrated significant improvements in the mean flexion arc, from 27° to 126° in 38 cases and from 26° to 126° in 46 cases, with satisfactory stability [29,30]. However, external fixation has drawbacks, including increased surgical difficulty, potential instability with improperly placed dynamic fixation, and longer operative times [31].

In light of advances in anatomic ligamentous constructs, the Internal Joint Stabilizer of the Elbow (IJS-E) has emerged as a dynamic internal fixator designed to address traumatic elbow instability, chronic dislocations, and augment fracture fixation [32]. Distinct from traditional methods, the IJS-E utilizes a hinged internal fixator that both provisionally stabilizes and maintains the elbow’s concentric position. The use of a hinged internal fixator was introduced by Orbay and Mijares using a Steinmann pin as an alternative approach to treat traumatic elbow instability. They achieved an average flexion–extension arc of 115° and pronation–supination arc of 139° [33]. Subsequent studies by Orbay et al. using IJS-E reported a mean arc of flexion and forearm rotation of 119° and 152°, respectively [34]. One of the IJS-E’s advantages lies in its ability to promote early, active elbow movement while ensuring anatomical alignment [34,35]. A potential drawback is the need for a subsequent device removal procedure. Despite these challenges, studies corroborate the efficacy of the IJS-E in addressing elbow instability. A more recent study by Pasternack et al. assessing postoperative functional outcomes in patients who received IJS-E for traumatic elbow instability reported mean flexion–extension and pronation–supination arcs of 106° and 141°, respectively, at final follow-up (average length 13.4 months). The average DASH and Broberg–Morrey scores were 28.7 and 68.2, respectively [32].

### 3.3. Limitations

This study has several limitations. First, its retrospective design and inclusion of two cases of iatrogenic elbow instability limit the generalizability of the findings. As a case series, the present study is intended to demonstrate the feasibility of internal brace augmentation in this clinical setting rather than establish definitive conclusions regarding its efficacy. Second, preoperative pain scores were not consistently recorded, precluding reliable assessment of changes in pain following surgery. Consequently, it is not possible to determine whether the observed postoperative pain levels were attributable to internal brace augmentation specifically or would have occurred following surgical intervention more broadly. Finally, the limited sample size and follow-up restrict the ability to evaluate long-term outcomes and complications associated with this technique. Future prospective studies with larger patient cohorts and standardized outcome measures are needed to further assess functional outcomes, pain, durability, and the overall effectiveness of internal brace augmentation for iatrogenic elbow instability.

### 3.4. Conclusions

Severe elbow contracture secondary to trauma often requires UCL release or excision, while iatrogenic valgus instability post-capsular release remains a challenging, unresolved issue. This paper presents the novel use of IB fixation to address valgus laxity, showing promising outcomes. However, further large-scale studies are necessary to evaluate its overall effectiveness and generalizability due to small sample sizes and heterogeneous follow-up.

## Data Availability

The original contributions presented in this study are included in the article. Further inquiries can be directed to the corresponding author.
